# Irrigation of the Colon

**Published:** 1883-01-20

**Authors:** Chas. W. Dulles

**Affiliations:** Surgical Registrar to the Hospital of the University of Pennsylvania


### IRRIGATION OF THE COLON.

By Chas. W. Dulles, M.D.,

Surgical Registrar to the Hospital of the University of Pennsylvania.

As we are now getting into that season when diseases of the intestines carry off the greatest number of victims, I desire to call attention to a method of treating inflammations of the colon, which has never—as far as I know—been at all generally adopted or even understood in this country; although it is not uncommonly practiced in Europe. It is not difficult or dangerous; on the contrary, it is simple and easy to carry out, and it cannot possibly do harm. The method was called by Dr. Alois Monti, of Vienna, whom I saw practice it often in 1876 and 1877, "irrigation of the large intestine."

It is carried out in the fnllowing manner: The patient being placed on the side, or back, or with the belly downward, and the pelvis a little elevated, a large, moderately flexible catheter, if for an infant or child—or a stomach tube, if for an adult—is inserted in the rectum. To this is attached, by a tube, a reservoir of water,* the heighth of which can be varied as may be required.

The water is now allowed to flow from a height of about two feet until the rectum is distended; meanwhile the end of the catheter or tube in the rectum is pressed gently but steadily upward toward the left iliac fossa, Very soon it will be found that the water has opened out the folds of the bowel and straightened the curves, so that the tube finds its way beyond the sigmoid flexure and into the descending colon. Unless the operator be very unskilful, it may now be pushed gently on, the flow of water continuing without interruption, until it reaches the left hypochondrium, when the transverse colon becomes the descending.

The flow of water is now to be continued until the whole colon, all the way to the cæcum, has been gently distended; the operator assuring himself of this by the amount of fluid used, and by palpation and percussion. The tube is now withdrawn and the operation is complete.

The fluid remains in the bowel a variable length of time. Sometimes it begins to come away in a few minutes, but it sometimes remains a half an hour or more.

This method I have seen used by Monti for various inflammatory disorders of the large intestine, as well as to cause expulsion of worms and flatus; and I have, myself, used it a number of times with results calculated to give me great faith in its usefulness.

The most striking case I now recall occurred in 1878, when I was summoned in the night to an infant a few months old, whom I found screaming and struggling with the pains of acute colitis. I took it on my knee, had cool water and a fountain syringe brought, attached the silver catheter from my pocket case, oiled it and slipped it first into the rectum and then up to the bend of the colon, and allowed about a pint and a half of water to flow in at that point. As the water filled the bowel the child's struggles and cries ceased, and it actually went to sleep before I was done, and onlv waked when the water began to be discharged.

Such striking results cannot be considered the rule, of course; but there can be no doubt that so complete a lavement must be of advantage in soothing the angry lining of the bowel and diluting and bringing away both the cause and the products of irritation.

To fill the outlines of the method a little, I will add that in gen-

---

*A fountain syringe, or any of its substitutes, serves this purpose well.

eral the fluid used should be cool, not cold water. It is rarely necessary to use astringents. When they are desired, the best is alum, in a one or two per cent. solution, with, perhaps, a few drops of laudanum added. The irrigations may be frequently repeated; and, in cases that do not get well promptly, various temperatures may be tried—from 70° or 80° to 40° Fahr.—depending on circumstances.

The amount of fluid to be used varies with the age of the patient. It should always be enough to fill the *entire* colon. An unweaned infant may require more than two pints, an adult several quarts.

No real syringe should be used if hydrostatic pressure can be obtained; though, if this is not to be had, I have found the syringe, carefully and slowly used, will serve very well.

Thus far I have referred mainly to such intestinal troubles as are most frequent in summer. The method is, I think, invaluable in all inflammatory affections of the colon, from diarrhœa to dysentery, and useful—for reasons I cannot go into now—in inflammations of the small intestine also.

Before leaving the subject, I want to speak of another use which I learned by experience last winter. I was called into the country to see a child about two years old, whom I found in convulsions. The use of revulsives had been tried without effect. I could get nothing in its mouth to produce vomiting or catharsis. The means at hand were very limited. I was satisfied from the history that the convulsions were due to irritating ingesta. I concluded to see if they were in the colon. So I took my silver catheter, attached it to a syringe, passed it through the anus, distended the rectum, pushed the catheter up till I could feel it through the abdominal wall, just below the lift costal cartilage, and filled the whole colon with warm water, in which a little soap had been stirred. After about three minutes the water came away and brought a mass of undigested and indigestible stuff that was quite sufficient to cause the trouble. The convulsions stopped and the child got quite well.

From this case, I think, a useful hint may be gathered, and I am sure I shall repeat my experiment the next time I have to treat a case of convulsions due to intestinal irritation.

I recall attention to this method because I think it too valuable to be allowed to be forgotten; and I hope that it may prove a helpful adjunct to our other therapeutic resources against intestinal disorders.—*Medical News.*

